# Method for mapping Hg^0^ emissions from gold shops in artisanal and small-scale gold mining communities

**DOI:** 10.1016/j.mex.2020.101060

**Published:** 2020-09-09

**Authors:** Samantha T. Brown, Kazi M. Hasan, Keegan H. Moody, Danielle C. Loving, Kathryn E. Howe, Alaina G. Dawson, Kevin Drace, Jeffrey D. Hugdahl, Caryn S. Seney, Claudia M. Vega, Luis E. Fernandez, Adam M. Kiefer

**Affiliations:** a Department of Chemistry, Mercer University Macon, GA, United States; b Department of Biology, Birmingham-Southern College Birmingham, AL, United States; c Centro de Innovación Científica Amazónica (CINCIA), Puerto Maldonado 17000, Madre de Dios, Peru; d Center for Energy, Environmental and Sustainability (CEES), Wake Forest University, Winston-Salem 27109, NC, United States

**Keywords:** Artisanal and Small-scale Gold Mining, Mercury vapor, Mercury, Gold Shops

## Abstract

Gold shops in artisanal and small-scale gold mining communities represent major point sources of airborne mercury pollution. Concentrations of elemental mercury (Hg^0^) emitted by these shops can be determined using a portable atomic absorbance spectrometer (AAS) with Zeeman correction. These measured Hg^0^ concentrations can then be correlated to position as determined by a hand-held GPS unit, and the resulting data mapped using a Geographic Information System (GIS). A detailed method for obtaining and analyzing data collected near gold shops in Mazuko, Peru is provided. Maps generated using this method were employed to identify point sources of Hg^0^ contamination originating from gold shops in ASGM communities and were shared with local city managers to assist in urban planning.

• A detailed method is provided to collect and process data, ultimately generating a map that allows for the screening of a community to identify point sources of Hg^0^ contamination.
• Raw data is provided, as well as a video detailing data processing and mapping using a common spreadsheet program and an open-source GIS.
• The generated map can be used for determining areas where people may be exposed to elevated Hg^0^ concentrations and/or occupational mercury vapor exposure, targeted enforcement, or outreach to limit Hg^0^ pollution.

## Graphical abstract

**Figure:**
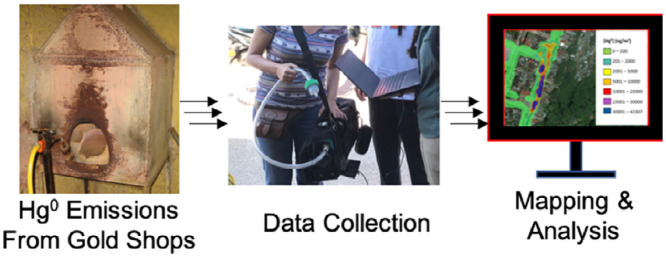

Specifications table

**Table:** 

Subject Area	Environmental Science
More specific subject area	*Air Mercury Pollution*
Method name	Method for Mapping Hg^0^ Emissions from Gold Shops in Artisanal and Small-scale Gold Mining Communities
Name and reference of original method	K.H. Moody, K.M. Hasan, S. Aljic, V.M. Blakeman, L.P. Hicks, D.C. Loving, M.E. Moore, B.S. Hammett, M. Silva-González, C.S. Seney, A.M. Kiefer, Mercury emissions from Peruvian gold shops: Potential ramifications for Minamata compliance in artisanal and small-scale gold mining communities, Environmental Research. 182 (2020) 109,042. 10.1016/j.envres.2019.109042.
Resource availability	*Lumex RA-915* *M, Laptop Computer, QGIS, GPS Unit, Mercury Instruments Mercury Tracker 3000 IP*

## Introduction

Artisanal and Small-scale Gold Mining (ASGM) is recognized as the largest source of anthropogenic mercury pollution to the atmosphere [1]. Miners add elemental mercury (Hg^0^) to amalgamate gold directly to the ore prior to concentration or to the concentrate, separating it from unwanted gangue minerals. The resulting solid amalgam is 40–60% by mass Hg^0^ when isolated and is heated (commonly referred to as “burned”) with an open flame to remove the majority of Hg^0^, revealing the sponge gold [2,3]. Because the heating process is inefficient, the sponge gold can still contain 2–5% Hg^0^ by mass [4,5]. Miners then take their sponge gold to a gold shop in a nearby community, where the sponge gold is reheated and a price offered for it by the shop owner. Some gold shops burn whole amalgams in the shop prior to sale. Unfortunately, gold shops are often poorly ventilated, and Hg^0^ emitted during burning is often vented into the shop and/or onto the street or sidewalk immediately outside of the shop. As a result, gold shops are the major source of Hg^0^ pollution in these communities and represent a threat to both human and environmental health [6], [7], [8], [9], [10].

The Minamata Convention on Mercury, an international treaty, was enacted to encourage signatory nations to decrease their reliance upon Hg and its related compounds [11]. Under the Convention, nations with significant ASGM activity must develop a National Action Plan specifying Hg pollution reduction targets for this sector, highlighting actions to be taken to eliminate the open burning of amalgams and sponge gold, particularly in residential areas [12]. Identifying gold shops and quantifying their emissions to the environment is problematic; however, utilizing portable spectrometers to map Hg^0^ concentrations in these communities has become a viable means of studying the impact these shops have on the surrounding community [8,[13], [14], [15].

Recently, a series of training programs and environmental assessments in Peru [8] have allowed for the formalization of a mapping technique that can be used to map ASGM communities over multiple days. This screening technique plots the maximum Hg^0^ concentration determined during the monitoring period at each unique set of geographical coordinates. The maps generated during this process can be used to streamline the quantification of Hg^0^ concentrations in and around these shops. Herein, we provide a detailed procedure for mapping Hg^0^ in ASGM communities, in addition to a video walkthrough and authentic data set to practice both the data analysis and generation of the map using a free geographic information system.

## Health and safety

Hg^0^ vapor is highly toxic, odorless, and invisible to the naked eye. It is often considered a chronic toxin, with exposure to concentrations as low as 20,000 ng/m^3^ over several years being implicated in damage to the central nervous system [16]. However, during the burning process Hg^0^ concentrations often greatly exceed 1,000,000 ng/m^3^ and miners have suffered debilitating lung damage and/or died from Hg^0^ poisoning [6], [7], [8],[17], [18], [19]. In ASGM communities with gold shops, Hg^0^ concentrations can change greatly depending on the proximity to the shop and whether or not burning is actively occurring. In areas of high [Hg^0^] concentrations, appropriate individual personal protective equipment including a reusable respirator with an appropriate filter should be available to those collecting data. Hg^0^ levels should be constantly monitored in real time to protect both those operating the instrumentation and the instrumentation itself. Since monitoring often occurs on streets and sidewalks, great care should be taken during monitoring to avoid vehicles and other traffic hazards.

## Materials and instrumentation

• Ohio Lumex RA-915 M Portable Mercury Vapor Analyzer (Lumex), calibrated by the manufacturer
• PC computer (Windows 10) compatible Ohio Lumex's *RAPID* software; Microsoft Excel for Office 365;^1^ QGIS v 2.18.24 with the Bing Aerial Tile Server and heatmap plugin;^2^ and Garmin Base Camp
• Garmin Oregon GPS Unit, or other GPS Unit capable of recording position (latitude and longitude) vs. time every second
• Mercury Instruments Mercury Tracker 3000 IP (MTIP) Hg^0^ vapor analyzer, calibrated by the manufacturer

## Preparation for data collection

The manufacturer of the Lumex lists the detection range of the instrument as being from 0.5 ng/m^3^- 50,000 ng/m^3^. However, the upper value of the calibration (provided in the calibration certificate) should be used, which in the present experiment was 42,307 ng/m^3^. The Lumex was operated using the Lumex manufacturer's *RAPID* software, and Hg^0^ concentrations were recorded every second. The computer time was synced to a GPS unit that was recording position in one second intervals. The following steps were completed sequentially prior to data collection.

• The MTIP, Lumex, GPS unit, and computer were brought to an uncovered, open area previously determined to have Hg^0^ levels < 100 ng/m^3^. Care was taken to ensure that the GPS unit was turned on, and there were no overhead obstructions ensuring that the GPS unit was able to link to the satellite.
• The MTIP was turned on and data collection started. Hg^0^ concentrations were confirmed to be below 1,000 ng/m^3^.
• Both the Lumex and the *RAPID* software were set to the following parameters.
  ○ The zero-correction time was set to 40 s.
  ○ The frame time was set to one second.
  ○ The monitoring duration was set to between 6 and 8 h regardless of how long data collection occured.
  ○ The zero check was set to every 20 min.
  ○ The alarm limit was set to 25,000 ng/m^3^.
• The Lumex was operated independently of the computer in an “On Stream” mode for 20 min prior to data collection. At the end of 20 min the relative deviation (*D* < 20%) was recorded.
• The time on the computer was manually synced to the GPS time. Team members synced their watches to the GPS time. The computer was connected to the Lumex using the appropriate cable.
• The *RAPID* Software was launched, air analysis monitoring was selected, and all parameters were confirmed. In order to start data collection, the play button on *RAPID* and the GPS are started at approximately the same time. The start time of the Lumex was recorded.
• The GPS Unit was directly connected to the Lumex carrying bag.

## Data collection in the field

Gold shops can emit concentrations of Hg^0^ exceeding 2,000,000 ng/m^3^, orders of magnitude higher than the upper limit of detection of the Lumex. The Lumex is prone to memory effects at high concentrations of Hg^0^. In addition, previous studies have demonstrated that the Lumex can itself become contaminated in communities where the burning of amalgams occurs, requiring repair and recalibration by the manufacturer [20]. To this end, a Mercury Instruments Mercury Tracker 3000 IP (MTIP) Hg^0^ vapor analyzer is used to measure Hg^0^ vapor concentrations in advance of the Lumex. The MTIP is very robust, capable of determining Hg^0^ concentrations ranging from 0 to 2,000,000 ng/m^3^. The MTIP precedes the Lumex by 3–15 m, and its operators verbally relay the location of elevated Hg^0^ concentrations to the operators of the Lumex. Using this information, the operators of the Lumex avoid these areas and thus the corresponding high levels of Hg^0^ vapor that could 1) pose a health risk to the operators themselves and 2) contaminate the Lumex. In addition to the dangers posed by elevated Hg^0^ concentrations, teams must be aware of their surroundings at all times to avoid vehicle traffic.

The Lumex is equipped with a ~1.2 m sampling probe through which air is drawn via an internal pump through the sample cell. As such, there is a short transit time between the air entering the Lumex and analysis. Lumex operators maintain a pace of ~0.75 m/s to compensate for this transit time. Although a previous study using a Lumex RA-915+ reported placing the Lumex in a vehicle during monitoring [14], we were unable to accurately measure Hg^0^ concentrations using the Lumex RA-915 M in a similar fashion. In addition, Hg^0^ concentrations decrease rapidly with increased distance from the source of Hg^0^ contamination and as such, monitoring was often conducted on sidewalks and areas where vehicles could not travel.

The following guidelines should be used during data collection by a mapping team of no fewer than five members:

• The MTIP is used to collect data in front of the Lumex to ensure the safety of the instrumentation and instrument operators.
  ○ A minimum of two people are required to operate the MTIP. One individual carries the instrumentation, while one watches for traffic and maintains a notebook. Both monitor the real-time Hg^0^ concentrations determined by the instrument.
  ○ The team walks ahead of Lumex team (3–15 m), monitoring Hg^0^ concentrations and informing the operators of the Lumex of elevated Hg^0^ concentrations.
  ○ The MTIP sampling probe is utilized at all times to ensure that the operator is removed from the area being sampled.
• The Lumex team is comprised of at least three individuals.
  ○ One person carries the Lumex/GPS unit and the connected sampling probe, which is maintained ~1 m from the ground.
  ○ One person carries the computer and ensures the cable connection between the Lumex and the computer remains intact. This person also sets the walking pace for the team.
  ○ One person is responsible for monitoring traffic and other potential safety hazards. This person is also responsible for monitoring the time period. Approximately 18 min after each baseline correction, this member directs the team to move to an area with low Hg^0^ concentrations for the next baseline correction.^3^
• During the data collection, all team members walk at a slow pace (~0.75 m/s, or 2.7 km/h) to ensure that the concentrations recorded are properly correlated to the location and to avoid entering areas of high Hg^0^ concentrations.
• Upon completing data collection, all raw data is saved. Lumex data is exported as a .txt file using the *RAPID* software. GPS data is saved to the device and downloaded to the computer as a spreadsheet using the Garmin BaseCamp software.

## Analysis of data and map generation

Upon completion of data collection, the data was processed using a spreadsheet program that contained both “MAXIFS” and “VLOOKUP” formulas. Both Hg^0^ concentration and position were recorded as functions of time, and as such, can be directly correlated through time. Each unique position was correlated to the maximum Hg^0^ concentration during the monitoring period, and these values were mapped using a GIS system. The resulting map displays the maximum concentrations of Hg^0^ at each location during the measurement period. It is important to note that this map reflects the Hg^0^ concentration at a given time, and thus can only be used as a screening tool to identify the location of point sources of Hg^0^ contamination in the air. When possible, mapping should occur over multiple days to ensure that the most accurate map is generated. The data provided in the supplemental information corresponds to seven experiments recorded over two days, and during previous studies in Peru data was collected over four days [8]. Data was not collected during rain. Previous studies in Peru were measured during rainy and dry seasons, and this techinique was able to identify and locate gold shops under all conditions based on Hg^0^ emissions [8].

The key components of data analysis and map generation are listed below:

• The Lumex Data is copied and pasted into an Excel Spreadsheet on a tab labeled “Lumex”. Other tabs are labeled GPS, VLOOKUP, MAX, and QGIS.
• Similarly, the time and location data from the GPS unit are copied and pasted into a tab labelled “GPS”.
• On the GPS tab, the time column is delimited to remove the “AM/PM” designation. Latitude and longitude are converted into decimal representations.^4^ These data points are then pasted into a new tab entitled “VLOOKUP”.
• On the “Lumex” tab the time are delimited to remove the “AM/PM” designation, and the time and Hg^0^ concentrations are copied into the “VLOOKUP” tab.
• While on the “VLOOKUP” tab, the VLOOKUP function is used to find an exact match between position and concentration based upon time. The position and products of the VLOOKUP function, which are the Hg^0^ concentrations at each position, are then copied into a tab labeled “MAX”.
• On the “MAX” tab, the concentration data are sorted smallest-to-largest and the selections are expanded to ensure that the position remains correlated to the concentration. All values that are recorded as “#N/A” and their corresponding location are removed from the data set.^5^
• On the “MAX” tab, a third column is generated containing unique position data (i.e. all duplicate positions are removed). Using the “MAXIFS” function, the maximum Hg^0^ concentrations at each unique position (latitude and longitude) are generated. The unique positions and corresponding concentrations are copied into the “QGIS” tab.
• On the QGIS tab, the position data is split into latitude and longitude and remain correlated to the maximum concentration for that position. This tab is then saved as a .csv file that can be uploaded into QGIS or another GIS system to be mapped.
• The .csv file is uploaded into QGIS and mapped. The minimum concentration is set to 0 ng/m^3^, and the maximum concentration is determined by the calibration of the Lumex RA-915M available during data collection.

A detailed, step-by-step video procedure is provided in the linked video, and data is provided in the supplementary materials to generate a map of gold shops in Mazuko, Peru.

## Method validation: mapping mercury vapor concentrations in Mazuko, Madre de Dios, Peru

The city of Mazuko is one of the principle urban centers in Madre de Dios, Peru, an Amazonian department with widespread Hg pollution and deforestation resulting from ASGM activities [21], [22], [23], [24]. We were asked by municipal government officials in Mazuko to map the portion of the InterOceanic Highway that passes through their town to (1) identify point sources of Hg^0^ vapor originating from gold shops, (2) preliminarily quantify Hg^0^ concentrations near these shops, (3) discuss potential health ramifications of exposure to Hg^0^, and (4) place our findings in the context of air quality standards for mercury [25,26]. The data would ultimately be used by the government officials to address public health concerns related to elevated levels of mercury originating from gold shops and assist local efforts to move existing shops to less populated and traveled areas of the city.

**Fig. 1:**
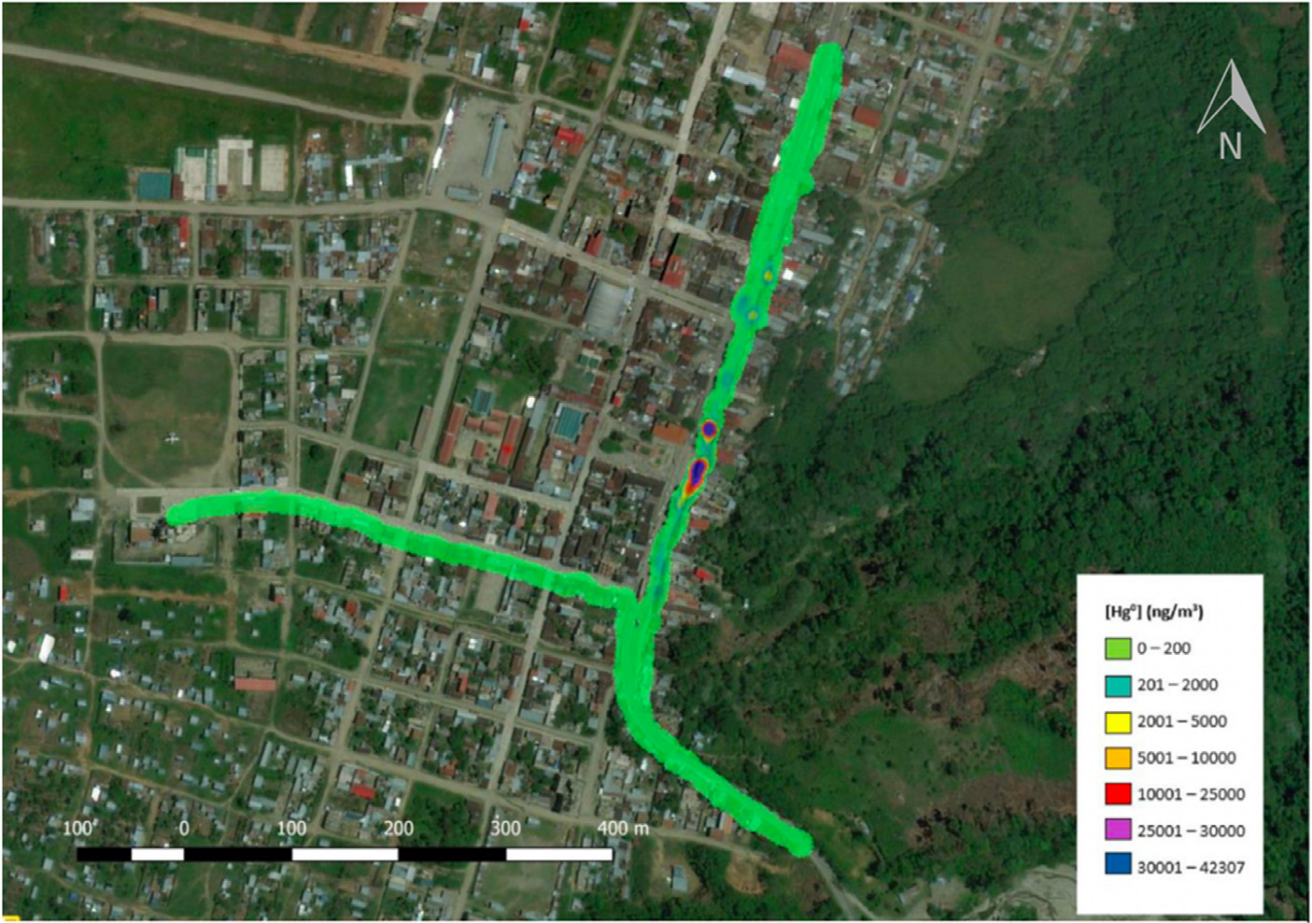
The map produced from the data provided in the supplemental information via the method reported here and in the video walk through.

Data collection was conducted on June 13 and 14, 2019 over the course of seven experiments. Upon completion of each experiment, a map was generated. Depending upon weather and other external factors, the number of experiments each day varied. When analyzed using the method described here and in the video, the data provided in the supporting information produced the map in Fig. 1. This map is representative of data collected during one experiment.

**Fig. 2:**
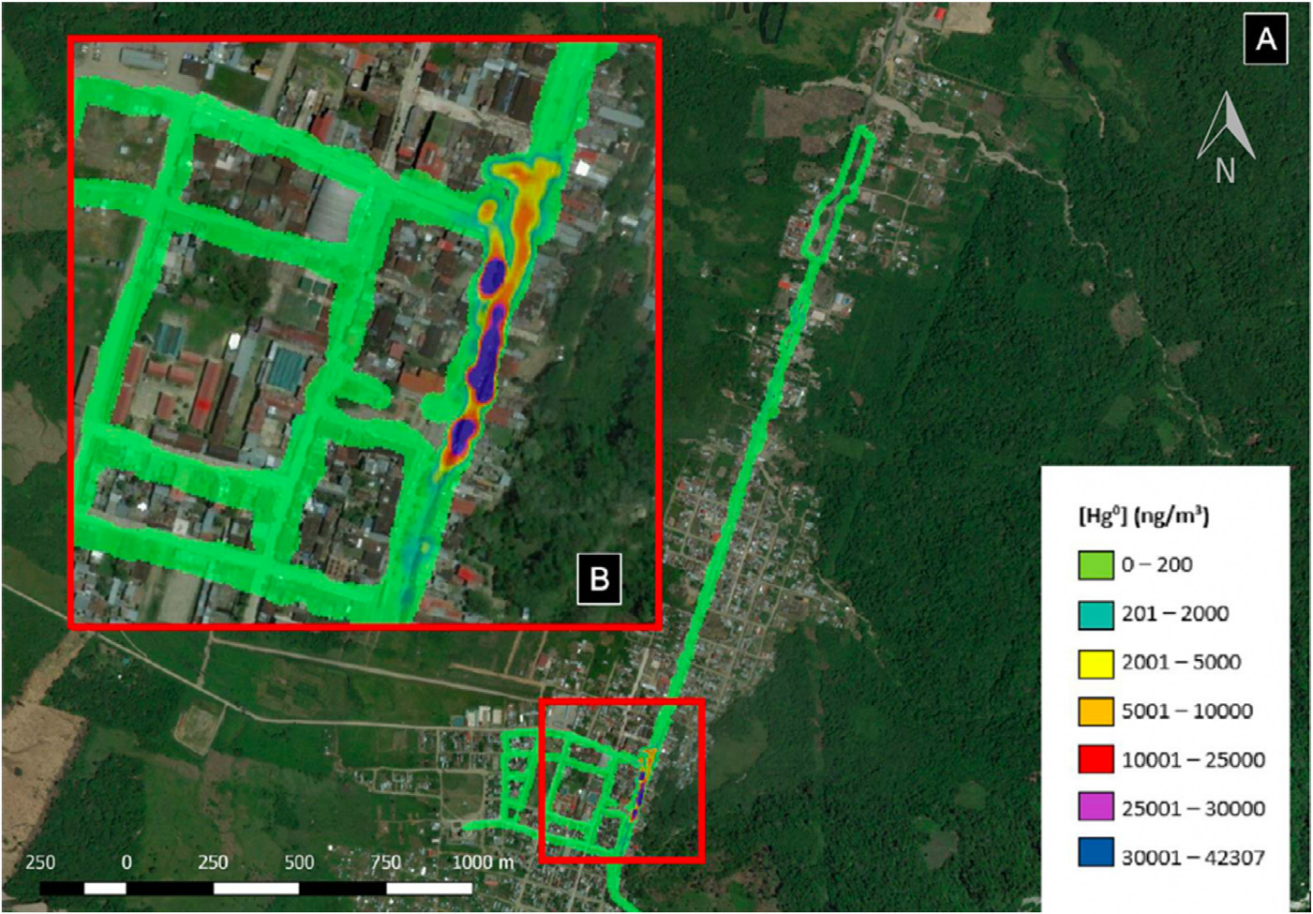
(A) Map of maximum Hg^0^ concentrations at each unique GPS position collected over seven experiments. (B) Enlarged portion of town highlighting location of gold shops. All Hg^0^ emissions were traced back to gold shops.

Fig. 2 is the map generated from all data collected during the seven experiments, recording the maximum Hg^0^ concentration at each unique latitude and longitude. All emissions of Hg^0^ in the study area were traced back to gold shops purchasing and burning amalgams and sponge gold. According to current Peruvian air quality standards for total gaseous mercury (TGM), at no time during a 24-h period should Hg^0^ concentrations exceed 2,000 ng/m^3^ [25,26]. Hg^0^ is a component of TGM, and is routinely exceeded in the vicinity of these gold shops. It is important to note that Hg^0^ concentrations often exceeded the values recorded by the Lumex by an order of magnitude as measured by the MTIP. In one case, the MTIP recorded concentrations more than 20 m away from a gold shop exceeding 1,000,000 ng/m^3^; to avoid contamination of the more sensitive Lumex, the Lumex team was prevented from entering the area.

## Limitations of the present method

Mapping using this method is a valuable tool for screening ASGM communities and identifying areas of high Hg^0^ concentrations associated with gold shops. However, it is a screening technique that only records the concentration of Hg^0^ at a given time. Gold shops that are shuttered, have tall chimneys for their ventilation, or even those that are significantly removed from the street may not be identified. To this end, it is essential that multiple experiments be conducted over multiple days to gain an accurate understanding of Hg^0^ concentrations. Even then, statistical analysis of data collected using this method is not recommended. Once the collection of data is complete, the resulting map can be used to guide the placement of more sensitive analytical instrumentation or passive air samplers (PASs) to measure either TGM or gaseous Hg^0^ concentrations over extended periods of time.

## Conclusion

The Ohio Lumex RA-915 M (Lumex) mercury analyzer can be used to determine Hg^0^ vapor concentrations associated with ASGM activities, and these concentrations can in turn be correlated with position (latitude and longitude) to map Hg^0^ concentrations throughout the community. The method presented herein allows for the generation of a map displaying elevated concentrations of Hg^0^ as a direct result of heating sponge gold and amalgams in ASGM gold shops. As a result, the maps can be used as a screening tool to identify major point sources of Hg^0^ emissions, particularly those stemming from the burning of amalgams, reheating of sponge gold, or smelting of sponge gold. There is a relatively short turn around time from data collection to generation of a map. The resulting map can be produced in the communities in which data is collected and immediately shared with the local government, gold shop owners, and other members of the communities. The maps can be used for the enforcement of health and environmental codes, and to inform the planning and construction of buildings such as schools and hospitals in which vulnerable populations congregate.

In addition, collected data can be used to inform future quantitative studies to estimate Hg^0^ emitted to the atmosphere in these communities, as well as the development of protocols for monitoring Hg^0^ emissions in accordance with existing air quality standards [8]. Moreover, the data can be used to guide follow-up monitoring, including but not limited to monitoring Hg^0^ concentrations inside gold shops, emitted from gold shop stacks, and 24-h time-weighted averages to determine compliance with local, regional, or national air quality standards. Current efforts are ongoing to compare this method with maps generated from PASs in Puerto Maldonado, Peru.

## Declaration of Competing Interest

The Authors confirm that there are no conflicts of interest.

## Appendix Supplementary materials

Image, application 1

Image, video 2

Image, application 3

## Footnotes

1 A version of Excel with VLOOKUP and MAXIFS formulas is required. Google Sheets may be used, although the procedure must be slightly altered during data work up.

2 QGIS Development Team (2020). QGIS Geographic Information System. Open Source Geospatial Foundation Project. http://qgis.osgeo.org.

3 Our group and others have noted that baseline correction of the Lumex should occur in areas of low concentration to ensure adequate operation of the instrument [8,14].

4 While some GPS units can record location data in decimal format, we have found that certain units provide less accurate locations using this setting. As a result, we provide the conversion of location data from degrees/minutes into decimals in the video.

5 The #N/A occurs as a result of starting or stopping the spectrometer before the GPS unit; it is a location without a concentration.
